# Editorial: Resources for developmental ecological psychology: organicism, epigenetics, relational development, dynamic systems

**DOI:** 10.3389/fpsyg.2026.1754164

**Published:** 2026-02-11

**Authors:** Agnes Szokolszky, Iris Nomikou, Lisette De-Jong Hoekstra, Catherine Read

**Affiliations:** 1 Doctoral School of Education, University of Szeged, Szeged, Hungary; 2 Centre for INTERACTION Development and Diversity, University of Portsmouth, Portsmouth, United Kingdom; 3 Department of Psychology, Faculty of Behavioral and Social Sciences, University of Groningen, Groningen, Netherlands; 4 Department of Psychology, Ithaca College, Ithaca, NY, United States

**Keywords:** bioecological approach, developmental dynamic systems approach, developmental ecological psychology, developmental phenomenology, developmental psychobiology, developmental relational systems approach, ecological-relational metatheory, sociocultural approach to development

## 1 Aims of the project and the meaning of “ecological”

With the Frontiers Research Topic “*Resources for Developmental Ecological Psychology*,” we aimed to bring together researchers from various branches of developmental psychology that can broadly be considered ecological. We sought to examine how these approaches relate to ecological ideas and to explore how researchers in the field apply ecological frameworks. Adopting an ecological approach involves using an ecological level of analysis. According to Ulric Neisser's broad yet programmatic definition: “The concept of an ecological level of analysis really includes every form of psychology that takes the physical or the social environment seriously” (Neisser, 1997, p. 96). “Taking seriously” means, with all its theoretical and methodological consequences, that a living being and its environment must be studied in their interconnectedness. This also means rejecting reductionist approaches to development, whether psychogenic or biogenic.

The ecological perspective implies that development is an interconnected, complex, and dynamic process involving organisms within their specific environments, their specific surroundings. Notable scientists such as John Dewey, Roger Barker, James J. Gibson, Eleanor J. Gibson, Gilbert Gottlieb, Kurt Lewin, and Lev Vygotsky worked out theories of development consistent with this broad view. From these foundations, several approaches have emerged over the past fifty years that, in their own ways, support the core idea of developmental ecology. Examples include Developmental Ecological Psychology, inspired by Gibson (1979/2014) and Gibson (1988, Gibson and Pick, 2000); the Bioecological Model by Bronfenbrenner (1979, 2001); Developmental Psychobiology developed by Gottlieb (1991, 2007); the Developmental Relational Systems Approach promoted by Overton (2015); Lerner (2006); Dynamic Systems Theory advanced in development by Thelen (1985); Thelen and Smith (1994); the Sociocultural Approach rooted in Vygotsky's work (1978; 1987) and extended by others, and Developmental Phenomenology, exemplified, for example, by Trevarthan's (1978) and Zahavi's (2008) work.

It is now widely accepted that the frameworks guiding theory and research in developmental science have shifted from Cartesian split-mechanistic paradigms to process-relational systems frameworks over recent decades (Budwig and Alexander, 2021; Lerner et al., 2014; Overton, 2013, 2015; Witherington and Lickliter, 2017; Witherington et al., 2025). This shift is due to the influence of the developmental approaches listed above. These approaches are metatheoretical frameworks that accommodate various theories and research practices. Although they mostly developed independently and emphasize different aspects, they generally align with ecological thinking, even if not explicitly labeled as such. In this context, the ecological perspective acts as a high-level approach or worldview.

Previously, we examined the emerging “coalition” of ecological–relational developmental approaches (Szokolszky and Read, 2018), and through this Research Project, we continue that exploration. First, we discuss core concepts of ecologically focused approaches: interaction/coupling and mutuality. Then, we review the influential theoretical frameworks mentioned earlier, which are also cited as theoretical backgrounds in the Research Topic's papers. Next, we introduce the papers, focusing on how they embody an ecological perspective.

## 2 The concepts of interaction/coupling, and mutuality

We can analyze ecologically oriented approaches by examining how they incorporate the theoretical ideas of interaction/coupling and mutuality into their ontological-epistemological frameworks. Interaction refers to any reciprocal influence or action between entities—such as organisms, variables, or systems. It highlights the exchange of effects. The concept of interaction suggests that the organism and environment exist separately but interact through exchanges that happen at specific moments. Contextualist approaches often adopt this view when exploring how individuals connect with the physical, cognitive-emotional, and socio-cultural aspects of their environments.^1^ The doctrine that behavior is the joint function of both context/situation (“outside”) and person (“inside”, including dispositions, agency, and intentionality) implies that potential causes of behavior can be classified into two exhaustive and mutually exclusive categories (Schweder, 1991). Preserving fundamental developmental dichotomies this way prolongs the Cartesian mechanistic worldview in developmental science.

The concept of organism-environment mutuality indicates a more profound interdependence or co-constitution, where each party's identity or function emerges through the connection itself (Dewey and Bentley, 1949; Costall, 2001; Gibson, 1979/2014; Overton, 2015; Read and Szokolszky, 2020). Mutuality ensures a core ontological and epistemological inseparability of the organism and its surround^2^. The organism and its evolutionary and developmental surround mutually shape one another; they are fundamentally interconnected. Mutuality encompasses interdependence, co-regulation, co-determination, and co-evolution in the connection between the organism and its surround. (Gibson 1966, 1979/2014) developed a theory of action and perception based on mutuality. He showed that in perceiving and acting, organism and environment do not need to be “conjoined,” especially not through some form of code or mental image (cf., Gibson, 1988, p. 256). This underpins the concept of direct perception, claiming that meaningful action is possible because the environment provides richly structured arrays that are directly accessible to the perceiving organism (Gibson, 1966, 1979/2014; Shaw et al., 1982; Still and Good, 1992; Read and Szokolszky, 2020). Mutuality offers a deeper ontological and epistemological basis for developmental science than the interaction framework because if organisms and their surround are fundamentally inseparable, the unit of development is the organism-in-its-surround, not an organism interacting with its environment. Pointing out mutuality as a foundational principle is all the more important, as it is rarely applied in psychology^3^.

### 2.1 Family resemblance of ecological—relational orientations in current developmental theories

Researchers (e.g., Budwig and Alexander, 2021; Witherington, 2007; Overton, 2007) have repeatedly argued that conceptualizations at the metatheoretical level shape every aspect of theory, research, and practice. Therefore, careful and critical attention to metatheoretical assumptions is crucial, as is aligning metatheoretical perspectives that share similar commitments. Despite their differences, the explicit or implicit ecological commitments of theories that can be considered as ecological—relational justify treating them as related by family resemblance (Wittgenstein, 1953/2009). They all contribute to ecological thinking in developmental science and provide theoretical reference points for research and further theorizing.

Next, we briefly review the theoretical developmental approaches we mentioned in the first section (see Table 1), focusing on how they contribute to ecological theory in developmental science, even when they do not label themselves as ecological.^4^ These approaches also serve as theoretical reference points in the collection of papers—a point we will revisit in the next section.

**Table 1:** Influential theoretical approaches, consonant with the ecological worldview.

**Theoretical approaches**	**Representative researchers**
1. Developmental Ecological Psychology	Eleanor J. Gibson, Ann Pick, Karen Adolph
2. Ecological Systems/Bioecological Model	Urie Bronfenbrenner, Stephen J. Ceci, Pamela A. Morris
3. Developmental Psychobiology	Gilbert Gottlieb, Paul Griffiths, Susan Oyama
4. Relational Developmental Systems	Richard M. Lerner, Willis F. Overton, Michael Lewis
5. Dynamic Systems Approach to Development	Esther Thelen, Linda B. Smith, Paul van Geert
6. Sociocultural Approach to Development	Lev Vygotsky, Michael Cole, Barbara Rogoff
7. Developmental Phenomenology	Colwyn Treverthan, Shaun Gallagher, Hanne De Jaegher

### 2.2 Developmental ecological psychology

Developmental Ecological Psychology was an early, pioneering, and influential alternative to Cartesian developmental psychology, building upon J. J. Gibson's ecological approach to psychology. Core to Ecological Psychology is the idea that perception results not from sensory stimulation, but from structured, specific, directly accessible, and meaningful ambient arrays for an actively engaged organism, tuned by its evolution, development, and situated intentions. Likewise, actions are meaning-oriented intentional acts of the organism as an agent and are not reducible to motor movements. This provides a basis for describing the mutual relationship of an organism and its surround in terms of concepts such as affordances and optic flow, which depend on direct perception. Developing agents actively seek and create new functional relationships within their structured social and physical surround because they can perceive the layout of surfaces and changing events around them. Infant development is driven by active exploration, which helps the child better detect invariant structures that specify affordances, relative to the child's body, skills, and intentions (Gibson, 1988; Gibson and Pick, 2000). This approach has generated valuable insights into key developmental questions in early childhood, such as perceptual learning, the role of affordances, and other topics (e.g., Adolph and Robinson, 2013; Dent-Read and Zukow-Goldring, 1997; Jacobs and Michaels, 2007; Heras-Escribano, 2019; Raczaszek-Leonardi et al., 2018; Szokolszky et al., 2019). Drawing from the ontology and epistemology of Ecological Psychology, DEP offers a mutuality-based theoretical framework for understanding the fundamental, inseparable aspects of development: action and perception. However, less attention has been paid within this approach to addressing issues related to overall organism-environment mutuality during life-course development. The concept of affordances still holds untapped potential for analyzing developmental experiences. As an emerging field, DEP needs to broaden its scope and methodology (Costall, 1995; Kiverstein and Artese, 2024; Read and Szokolszky, 2018, 2024).

### 2.3 The ecological systems/bioecological model of human development

In his early ecological approach, Bronfenbrenner (1979) influenced by Roger Barker, described systems of environmental influences at increasingly complex levels—micro, meso, exo, and macro—as multiple layers of context that affect human development throughout life. This framework emphasized mapping contextual influences on development, viewing them as engagement embedded within existing systems. Later, Bronfenbrenner (2001) expanded his theory into the Bioecological Model by including four defining properties: process, person (biological and genetic traits), context, and time (chronosystems). Between the 1970s and 1990s, this approach was known as “Ecological Systems Theory.” The updated and final framework is called the “Bioecological Model of Human Development” (Bronfenbrenner and Morris, 2007). Development is seen as resulting from the reciprocal influence of the model's contextual factors. The model incorporates many aspects of related perspectives, such as lifespan psychology and cultural theory. Its distinctive feature is an interdisciplinary and integrative focus on childhood and adolescence, along with an interest in applications to policies and programs that promote youth and family development (Bronfenbrenner and Morris, 2007). The model sparked extensive research, though initially it faced criticism for being mostly descriptive and static, and for assuming passivity in the developing individual. Though regarded as more aligned with developmental systems approaches, the bioecological model was still viewed as lacking in dynamics and theoretically underdeveloped (Lerner, 2006). It was also critiqued for lacking an ecological stance on ontology and epistemology (Szokolszky and Read, 2018). Additionally, many studies cite the model but only use it superficially, focusing solely on specific contextual variables (Tudge et al., 2009).

### 2.4 Developmental psychobiology

The psychobiological systems approach, originally developed by Gottlieb (1971, 1991, 1992, 2007), played a key role in moving beyond genetic determinism, mechanistic environmentalism, and the nature-nurture dichotomy. Drawing on biological systems theories, Gottlieb offered a comprehensive framework centered on probabilistic epigenesis: the idea that development is not a predetermined process but an emergent, “fully coactional” process, jointly influenced by organismic factors (genetic, epigenetic, neural, behavioral) and typically present environmental influences. Developmental causes are systemic and relational; they operate at multiple levels (genetic, neural, physiological, and social) and involve coaction within and across these levels. This framework explains both the flexibility and robustness of development: in normal development, the ontogenetic niche reliably provides all necessary experiences, but local circumstances can significantly influence development, often in non-linear ways. Each individual develops along unique paths and with distinct histories. Experience is emphasized as a developmental factor that impacts gene expression and neural pathways within the interconnected system; it is a product of evolution marked by developmental plasticity. The dense, interconnected, and mutually constraining nature of development is illustrated through the concept of the developmental manifold—an intricate web of bidirectional relationships in a linear flow from past to present. Novelty and organizational complexity arise from the entire developmental manifold over time. Three crucial ideas are the importance of the organizing principle of integrative levels, the tendency toward increased complexity with evolution, and the contextual nature of behavioral events (Gottlieb et al., 2006; Greenberg et al., 2006). This approach firmly established a systemic, relational, experience-based view of development. At the same time, it was noted that this approach fails to regard the organism as a distinctly formative agent and also that defining experience as “the functioning of the senses” places this theory in the sense-based approach to perception/action/cognition, excluding an ontology grounded in mutuality (Read and Szokolszky, 2020, 2024).

### 2.5 Relational developmental systems

Relational Developmental Systems, as a high-level theory, was proposed by (Overton 2007; 2013; 2015; Overton and Lerner, 2014). It was conceived as an integrative framework for all approaches that aim to replace the old Cartesian-Split-Mechanistic Worldview. It drew from biological systems thinking as well as David Magnusson's person-oriented holistic-interactionist model of development, according to which the person is not a set of traits but a functionally organized whole (Magnusson, 1988; Magnusson and Stattin, 2006).

Based on process ontology (ontology of becoming), development is viewed as an emergent property resulting from ongoing, reciprocal, and dynamic interactions between individuals and their contexts over time, with the organism considered the producer of its own development (Lerner, 2021). Advocates of the RDS approach oppose mechanistic contextualism, which reduces action to motor activity. Instead, they support organicism-contextualism, where ‘act' pertains to the goal-directed functioning of a system (Overton, 2007; Overton and Ennis, 2006). The individual's contextual relationships are the fundamental unit of analysis in human development. Reciprocally bidirectional, synergistic, or fused relational processes form the basis for individual action and growth (Overton and Molenaar, 2015). Positive Youth Development—a theoretical branch—builds on developmental plasticity and highlights positive change in personal development; when young people are placed in circumstances that promote positive development, their attributes can change positively through their interactions within the relational developmental system (Lerner, 2018, 2021). A broader aim is to optimize the course of human development in applied settings. Witherington and Margett (2019) argue that the RDS approach is a vital and integrative theoretical model. However, it is presented at a highly abstract conceptual level and offers limited methodological strategies for research implementation.

### 2.6 The dynamic systems approach (DSA) to development

Developmental thinking based on the dynamic systems approach (Kelso, 1995; Van Geert, 1994, 2020) aims to explain the novelty, regularity, and orderliness of behavior using the concepts of self-organization and emergence. The core idea is that behavior, and overall development, are self-organizing, non-linear processes that arise from continuous interactions among the traditional categories of biological, psychological, and environmental factors over time. New developmental patterns emerge spontaneously through real-time interactions and coordination within the system, with development happening through shifts and phase transitions (Thelen and Smith, 1994, 2006; Lewis, 2000). Research within this framework primarily focuses on motor coordination (e.g., Thelen and Spencer, 1998), motor development (e.g., Adolph et al., 2014), and learning dynamics in various psychological domains such as cognitive development (e.g., De Jonge-Hoekstra and Cox, 2020; Stephen et al., 2009), language development (e.g., Cox and van Dijk, 2013), identity development (e.g., Van der Gaag et al., 2015), talent development (e.g., De Hartigh et al., 2018), and teacher-student interactions (e.g., Menninga et al., 2019). The focus is on time-dependent changes within the system, analyzed through computational modeling or non-linear timeseries analysis. The significant contribution of DSA has been to provide innovative methods, along with a formalized theoretical approach to development as a process of dense, nested interactions (coactions) unfolding in time. These methods have also been adopted by researchers using other frameworks, such as Ecological Psychology. DSA has renewed the multi-level contextualist theoretical framework, making self-organization a practically applicable concept and a broad research framework. DSA serves as a valuable overarching theoretical framework for understanding stability and change in development. However, Witherington (2007) distinguishes two main groups within this framework with distinct worldviews. The “pure contextualist camp” emphasizes the present moment when explaining development, viewing movement and behavior as embedded in specific tasks, and it tends to slide into mechanistic principles, overlooking the organism as an integrated whole. Conversely, the “organismic contextualist camp” considers both the local context and the organism's higher-order structure in its explanations (Lerner, 2006; Overton, 2007; Witherington, 2007; Witherington and Lickliter, 2017). Therefore, dynamical systems approaches differ, and thereby raise questions, about how they conceptualize the ontology of development.

### 2.7 The sociocultural approach

Vygotsky's (1978, 1987) theoretical influence was rooted in his core idea that higher mental functions develop through social interaction and cultural tools like language. His focus was on cognitive processes such as verbal thought, intentional recall, and problem-solving. Since the 1990s, a broad understanding of the cultural-historical nature of development has emerged along these lines. The sociocultural roots of the mind have become a central focus of research. Cole (1996) described development as participation in culturally organized practices. Valsiner (1998) connected sociocultural theory with developmental systems and phenomenological perspectives. Research on guided participation in cultural practices has redefined development as participatory transformation within such practices (e.g., Lave and Wenger, 1991; Rogoff, 2003, 2008). The aim in this framework is to understand how children learn to engage in the complex range of sociocultural activities and how different cultures introduce children to various practices at different developmental stages. Sociocultural approaches have clarified that development, with its multiple pathways, is rooted in culturally specific environments. They have introduced new themes and concepts, including guidance and participation in collective practices, apprenticeship learning, and cultural canalization. It is now widely accepted that learning and development are inseparable from the cultural, historical, and social contexts in which they occur. Recently, researchers have become more interested in examining developmental phenomena from cross-cultural perspectives. There is a shift from viewing culture as a fixed set of values and practices—defined as independent variables—to seeing it as continuously created and reshaped as people question, adapt, and redefine their values and practices (Misra and Babu, 2013). Sociocultural research relies on detailed, context-specific ethnographic data but does not specifically articulate cultural mediation and developmental change. It has also faced criticism for neglecting biopsychological processes, underestimating individual differences and personal agency, and assuming perception is a limited way of knowing (Toomela et al., 2014; Dent-Read and Zukow-Goldring, 1997). Furthermore, some argue that sociocultural research can fall into a dichotomy between the person/mind and culture (Schweder, 1991).

### 2.8 Developmental phenomenology

Developmental Phenomenology is an approach that combines the study of lived experience with developmental psychology to understand how a person's subjective experience changes and evolves over their lifetime. An important insight of phenomenology, most notably expressed by Merleau-Ponty (1962/1945), is that in human practice, the world appears to shared awareness before any conscious reflection. Intersubjectivity based on shared, embodied, direct awareness is a fundamental concept in phenomenology. Building on Merleau-Ponty's argument, modern phenomenology views the developing individual as an embodied, intentional, meaning-making whole (Gallagher, 2008; Fuchs and De Jaegher, 2009). Lived experience is studied from both the first- and second-person perspectives. Interaction is examined as “participatory sense making,” where meaning is created and transformed within the interaction itself (De Jaegher and Di Paolo, 2007). Theorists suggest that intersubjectivity is rooted in intercorporeality, meaning perception–action loops of mutual bodily responses and shared bodily awareness (Gallagher, 2008; Fuchs and De Jaegher, 2009). Sense-making is an ongoing engagement with the world, and developmental phenomenology seeks to understand the origins of this experience (Vincini and Gallagher, 2021). Research guided by this theory has demonstrated that infants possess an immediately responsive, conscious awareness of adults' communicative intentions, called primary intersubjectivity (Kokkinaki et al., 2023; Trevarthan, 1978; Sorjonen et al., 2021). Developmental Phenomenology assumes (with Phenomenological Psychology) that most fundamental access to other minds is direct and perceptual (Gallagher and Varga, 2014) and seeks to explain this direct social awareness. Representatives of this approach assume a sensation-based perception and consider direct social awareness arising from the assimilation and accommodation of configurations of sensory experience, combined with innate perceptual abilities and needs (Vincini and Gallagher, 2021). From a relational perspective, it was noted that the descriptive focus on subjective and intersubjective experience, along with the oversight of biological and cultural contexts, limits explanatory power (Overton, 2015; Witherington and Margett, 2011). Additionally, sensation-based explanation excludes a mutuality-based understanding of organism-surround relations (Read and Szokolszky, 2020).

Next, we turn to the papers in this Research Topic to review them, focusing on how the authors interpret and methodologically realize ecological perspectives.

## 3 The papers in this Research Topic

Our call for papers stated: “The article collection focuses on the different ways ecological thinking (broadly defined) has been integrated into developmental psychology, and the various ways ecological ideas can connect areas of developmental theory and research.” Authors responded by showcasing a range of empirical research, review papers, conceptual analysis, and theoretical papers. Some of the authors tied their research to a particular theoretical framework, others integrated ideas from multiple frameworks, and still others did not specify a theoretical background but, in their design and analytical methods, used resources that can be considered ecological in a broader sense. In what follows, we outline the articles, paying attention to how the authors interpret the meaning of ecological, how they implement it where relevant in their empirical investigations, and in what sense and to what extent the work fits the ecological perspective.

In this Research Topic of papers, two groups of authors connect their theoretical backgrounds to Bronfenbrenner's Bioecological Model. In their paper “Preschoolers' Prosocial Behavior in Groups—Testing Effects of Dominance, Popularity, and Friendship,” Katerkamp and Horn examined preschoolers' prosocial behavior as the result of a complex interaction among proximal processes, individual characteristics, environmental context, and time. While prosocial behavior is a well-studied topic, most research has relied on controlled laboratory experiments testing children alone or in pairs. This paper is therefore ecologically mindful, as the authors studied prosocial behavior in children's natural social environments, within group settings, and during interactions with familiar social partners. The authors focused on how dominance, popularity, and friendships interplay as key factors influencing prosocial behavior, treating these variables as interacting rather than as co-constructing one another. A sociometric assessment of popularity and friendship within the peer group was followed by three field-experimental sessions involving age-appropriate games designed to elicit dominant and/or prosocial behaviors in the groups. Finally, measures of children's popularity, friendships, dominance, and prosociality were externally validated using a teacher questionnaire. Data analysis used standard linear statistical techniques, with correlations at its core, suggesting that selected contextual factors are static and interact with each other. The study underscores that understanding children's prosocial behavior requires a multifaceted ecological approach. It also highlights a future challenge: the methodological and data analysis complexities involved in applying a dynamic, ecological theoretical framework.

Aslanova et al. in “Does sibling family structure matter in the emotion understanding development in preschoolers?” also place their study in the context of Bronfenbrenner's Bioecological Model, in which the child is seen as developing in various levels of social systems, from family to community to institutions, and constantly interacting with her social environment. The authors present a study of how preschoolers' understanding of emotions develops from age five to age six, in relation to contextual variables in family structure: number of siblings, age gaps, presence of a twin, sibling position, and gender composition. While the study has a longitudinal design that can detect changes in the system, the participating children were not observed in interaction or in their family settings. The authors study this topic using a standardized test, the Test of Emotion Comprehension, adapted for research in Russian. The design included three additional standardized tests to evaluate cognitive flexibility, visual and verbal working memory, and non-verbal intelligence. These test scores were then analyzed using non-parametric tests to examine relationships between test scores and family structure variables. The method employed in this study is a traditional psychometric approach to the topic. The authors seem to use the ecological paradigm as a point of departure for their research, rather than a methodological approach. They acknowledge that more dynamic and ecological measures and observations would expand these results in important ways. The important contribution of this research lies in the authors' efforts to expand the typical use of cognitive, standardized, and individualistic measures into broader social contexts.

Gavrilova and Kornienko's research paper, “Perezhivanie as a Source of Children's Development: Case of Emotional Development Intervention through Visual Arts,” builds on Vygotsky's sociocultural theory to explore children's emotional experiences through a case study involving a sibling pair. The authors base their research on Vygotskyan concepts of “perezhivanie” and “refraction”—terms currently used to include emotional aspects in educational research and to support personalized learning. Perezhivanie, described as “a unit of consciousness,” refers to the personal significance and emotional impact of a specific developmental experience, while refraction suggests that the child internally interprets this experience. This research paper embodies the “ecological” perspective by employing an innovative qualitative method; the authors created a dialogical setting in which the child and the researcher viewed and discussed an emotionally charged painting during individual sessions. The following day, the children drew and described their memories of the painting. Video analysis focused on verbal responses, behavioral cues, and features of the drawings, seeking signs of perezhivanie in the child's experience. The authors found that, while “refracting” the experience, the siblings responded differently to the same painting due to various developmental factors, including influences from their socio-cultural family environment. The authors present their research as an attempt to capture “the complexity and multidimensionality of the relationship between environment and psychological processes”. They note that the concept of perezhivanie poses a significant challenge for researchers due to its variable interpretations and empirical difficulties (see also Christodoulakis et al., 2021; Smagorinsky, 2011).

In their paper “A Scoping Review of the Research Evidence of the Developmental Assets Model in Europe,” Martin-Barrado and Gomez-Baya used the Web of Science database to provide an in-depth review of articles published between 2013 and 2024 in Europe that address the Developmental Assets model. The model is aligned with Relational Developmental Systems as a metatheory and Positive Youth Development (PYD) as a theoretical framework, widely used in the United States to study the transition from adolescence to adulthood. Research in the PYD model found strong evidence for the importance of five interrelated components (the 5Cs): Competence (positive self-concept in different areas), Confidence (positive self-worth), Connection (positive relationships with others), Character (respect for cultural and social values), and Caring (sympathy and empathy for others). The DA model further describes how personal and contextual resources (internal and external assets) jointly contribute to the positive transition from adolescence to adulthood. The authors therefore see as “ecological” the interaction of internal and external resources. Creators of this research model identified 20 internal and 20 external assets, based on the Developmental Assets Profile, introduced as a measurement tool (Benson et al., 2004). Results of the scoping review showed that the evidence for DA in Europe was consistent with North American research: a higher presence of developmental assets was associated with higher wellbeing, better psychological adjustment, and lower risk behaviors. A common conclusion of this literature is that positive development occurs when there is an alignment between internal strengths (e.g., positive future expectations) and contextual assets (e.g., social support). Evidently, both the PYD and the DA models capture important, well-researched components of youth development and inform intervention practices. At the same time, the asset perspective reduces the developmental processes it examines to the interaction of predetermined internal and external factors, thereby falling short of the principles of the relational developmental systems paradigm, which aims to capture the complex system of reciprocal influences in development.

The Dynamic Systems Approach, combined with the notion of affordances (defined as possibilities for action) is represented in the theory article “Cognitive Understanding is a Functional Coordination Pattern, Just Like Swimming.” Here, de Jonge-Hoekstra argues that all human and animal movement can be understood as arising from a dynamic system that self-assembles, including the gestures children make when answering questions about ‘cognitive' problems, such as the location of the balance point of a rigid bar. The argument, therefore, draws on dynamic systems theory as used in research on the development of skilled movement, with the added idea that children can perceive what the surroundings afford (in line with Gibson's (1979/2014), work on perception). The author, interestingly, extends her argument beyond skill development to a cognitive task involving conversation, perception, and gesture/action on the part of the adult and the child. She points out that previous research on such ‘cognitive' abilities as Piaget's concept of object permanence has shown that the ‘cognitive' ability is sensitive to the particulars of the surrounding surfaces (see also, Rader et al., 1979), and, therefore, it is important carefully to study the actual situation and actions involved in ‘showing knowledge'. If cognitive abilities are seen as arising from systems that self-assemble given the present task and surroundings, several problems with mental representation are avoided, and skilled action and cognitive ability are aligned within a single, consistent theoretical approach. This article, therefore, presents an interesting comparison with Aslanova et al., who call for efforts to make cognitive tasks more “ecological” by involving the child's environment. In this approach, the whole of the organism-in-its-surround is cognition. This paper is “ecological” in the sense that it analyzes and values the physical and social surroundings of the children whose development is being studied. The transfer of ideas and methods from research on skilled action to studies of cognitive tasks has the potential to provide a completely new understanding of how children know, and how they show their knowing.

In contrast to previous papers, Cornejo et al., in their paper “Spontaneous Bodily Coordination Varies Across Affective and Intellectual Child-Adult Interactions,” do not draw from a particular theoretical framework. The authors investigated symmetrical and asymmetrical bodily coordination between children and a non-familiar adult during experimentally manipulated storytelling situations. The theoretical background draws on multiple sensitizing developmental and interpersonal coordination concepts, such as mirroring, synchronization, and complementary actions, which allude to dynamical systems, but the paper does not pursue their systematic integration into a unified theoretical framework. The study can be considered “ecological” because it focuses on how naturalistic factors, such as affective and intellectual story content and narrating, shape bodily coordination in real-time social interactions. The authors' use of “ecological” thus refers to the influence of the social and physical context on children's behavior (more interaction-based), rather than to mutual reciprocity between children and their environment. While the experimental design richly captures spontaneous, social interaction-based behavior and contributes valuable data, the application of “ecological” does now go deeper than the design.

Similarly, in their paper “Advancing the Understanding of Children's Digital Engagement: Responsive Methodologies and Ethical Considerations in Psychological Research,” Kucirkova et al. review various research approaches for capturing children's perspectives. The authors consider the field of digital media engagement as an example of a dynamic field that requires research methods capable of capturing active engagement with technology. At the same time, they evaluate these approaches with reference to ethical principles for conducting research. The paper does not explicitly depart from a specific ecological theoretical framework, yet the methods presented include elements compatible with a hybrid theoretical approach. Firstly, the authors mention methods for capturing the immersive, dynamic, situated experience of children's engagement with media, which they term “responsive methodologies”. Consistent with interaction-based ecological approaches, they argue for capturing real-time interactions between child and technology. There is also mention of the notion of affordances of technology and the opportunities for action they provide. Finally, the authors discuss the relevance of nested systems of influence, such as the ones within the Bioecological Model. The authors present a compelling comparison of the benefits of employing both contextualized and decontextualised (reflective) methods to provide more ecologically valid, comprehensive explanations of developmental phenomena. Overall, this paper uses concepts from ecological approaches as a guide to research and an explanatory framework, without connecting to the assumptions and principles to an ecological theory of development.

In their paper “Developmental Ecological Psychology Meets Organicist Biology: The Example of the Ecological”, Read and Szokolszky aim to integrate Organicism and Ecological Psychology into a coherent framework of Developmental Ecological Psychology. The paper discusses, compares, and integrates several theoretical perspectives: Ecological Psychology, Dynamic Systems Theory, developmental models from Waddington and Gottlieb, and Organicism. The organicist approach highlights the life course of organisms as living at interrelated levels of functioning that influence each other both from the bottom up and top down, with forms and structures emerging from these dynamic interactions. The organicist approach thereby shows clear parallels with the other discussed perspectives. Rather than adhering to a single theoretical framework, the paper connects ecological, developmental, and organicist theories around the central concept of the acting and perceiving organism and its agency. The work is “ecological” in a deep sense: Development is understood as the continuous mutuality between organism and environment across the life cycle, and emphasizes that agency, action, and perception are inseparable. The authors interpret ecology as grounded in organism–environment mutuality based on direct perception. The paper thereby offers a rich, life-span view of development as an ongoing organism–surround process. While conceptually dense and occasionally demanding to read, the paper makes a strong contribution to unifying ecological and developmental theory and thereby achieves a profound application of ecological thinking, with open questions of empirical realization.

Similarly, Kärtner and Köster propose an integrative approach in their paper “Early social-cognitive development as a dynamic developmental system—a lifeworld approach.” The authors present a proposal for conceptualizing and studying early social and cognitive development. The lifeworld approach is deeply grounded in psychobiological and dynamical systems theories and offers a hybrid framework for examining variation within and across groups, aiming to understand internal and external influences within the context of social interactions that may explain development as a complex, non-deterministic process. The authors provide a hypothesis and review existing tools to explore how internal and external forces, as well as people, change through mutuality-based co-regulation at the behavioral level. It thus seeks to explain internal factors via external behaviors. Similar to de Jonge-Hoesktra, the authors situate social cognitive development within the world but focus more on social relationships. An interesting contribution of the paper is its conceptual framework for addressing culture-specific development. This area has not been a primary focus of research within these theoretical paradigms. It supports a variety of analytical methods that allow the analysis of the dyad as the unit of study, thus offering explanations that incorporate the environment in which infants develop.

Finally, in their paper “Understanding Identity Development in Context: Comparing Reflective and Situated Approaches to Identity,” Van der Gaag et al. offer a comparative view on how identity develops. Specifically, starting from the idea that social and historical contexts shape identity development, they contrast what they call “reflective” and “situated” approaches to understanding the influence and role of these contexts. The authors show how these common perspectives can differ significantly due to their distinct theoretical bases. These differences also lead to varied research methods. The authors use a hybrid theoretical model that combines developmental ecological psychology with complex dynamical systems theory. They see the person and their changing environment as co-creating each other, forming one person-environment system. This view aligns with phenomenological qualitative approaches that analyze actions in everyday settings. They also integrate principles from complex dynamic systems. Accordingly, the individual and social-cultural contexts constantly interact, creating a dynamic landscape of identity in which identity is not fixed but depends on various contexts that serve as constraints. In an ecological vein, the paper emphasizes the inseparability of person and environment at its core. The social-cultural context is not just an external factor eliciting emotional and evaluative responses leading to identity formation, but is an integral part of identity itself. The authors also explore how different theoretical approaches influence research design and suggest a pluralistic approach that allows different frameworks to work together to deepen our understanding of identity development.

## 4 Conclusion

The papers in the Research Topic offer a broad and flexible perspective on the meaning and application of an ecological approach to theory and research. There are notable differences in how the ecological worldview is understood and utilized in both theory and practice. “Ecological” is more often interpreted within an interaction framework than within a mutuality framework. Theoretical papers demonstrate the difficulty of integrating theories coherently with clear methodological applications. Empirical papers highlight the challenge of translating complex metatheories and their derived theories into practical research methods. It has been observed that, in research papers, there is often a gap between the theoretical foundations cited and the actual research methods employed, and elements of the mechanistic paradigm still dominate. Researchers tend to examine specific linear effects of individual or contextual factors on outcomes, often treating these factors as independent of context (Budwig and Alexander, 2021; O'Brien, 2005; Tudge et al., 2016). However, these problems do not concern solely individual researchers. Translating ecological–relational theoretical frameworks into research practice poses a challenge for developmental science as a whole (Molenaar et al., 2014).

We must recognize that “ecological” is an increasingly popular, often vaguely used term within the developmental field. Neisser's advice to “take the environment seriously” (Neisser, 1997) can provide a common ground, but this should not justify the superficial use of the term. Approaches rooted in strong ecological foundations should go beyond specific research topics or subfields. A comprehensive ecological-process-relational developmental theory should guide the field and address questions about development as the process of becoming across different timescales and multiple contexts throughout the entire lifespan. This includes a new understanding of the role of biology in human development, based on an integrated view of evolution and epigenetics (Lerner et al., 2014; Overton, 2015; Read and Szokolszky, 2024).

As Overton (2007) and Witherington (2007) observed, clarifying the metatheories operating within any field is essential, as they serve as the foundation for consistent and coherent theorizing and research practices. Describing the current shift in developmental theory as “process-relational” overlooks the critical aspect of “ecological.” The metatheoretical change has not only involved adopting an ontology and epistemology based on processes and relations, but also on ecological organism-environment processes. In this context, we emphasize the importance of developing explicitly ecological-relational metatheories that foster greater cohesion among contextualist, dynamic, and systems-based approaches in the field, as well as optimal developmental interventions in applied settings. And we point out the challenge of combining approaches that make contradictory metatheoretical assumptions, specifically regarding the organism-in-its-surround, seen either through mutuality (direct perception) or disjunctive interaction.

## Footnotes

1 Please note that here we use the term “interaction” in a strict sense, as the interaction between disjunct context/situation and person. In many scientific papers the term “interaction” is used to refer to what we call “mutuality” in this paper. These usages entail, however different metatheoretical assumptions, awareness of which is important.

2 Within the mutuality approach we prefer to use the words “surround” instead of “environment”, which is a term that is too broad and abstract. The term “surroundings” refers to the embeddedness of the perceiving-acting organism in the sense of mutuality, incorporating the inseparable, dynamic totality of factors present in the existence of the organism.

3 Another use of the word is mutuality as a quality of dyadic human relationships. In “mutual intersubjectivity” one is both affecting the other and being affected by the other; one extends oneself out to the other and is also receptive to the impact of the other” (Jordan, 1986, p. 2).

4 These approaches, along with historical background, are discussed in more detail in Szokolszky and Read (2018).
